# Mature Students’ Experience: A Community of Inquiry Study during a COVID-19 Pandemic

**DOI:** 10.1177/14779714221096175

**Published:** 2022-11

**Authors:** Damien Homer

**Affiliations:** 2707 University of Warwick, Coventry, UK

**Keywords:** mature students, blended learning, higher education, community of inquiry

## Abstract

The COVID-19 pandemic has had a profound effect on university students across the world. In a short period of time from 2020 to 2022, Higher Education Institutions (HEIs) had to pivot their modes of delivery to ensure they could meet the needs of their students. The move to digital platforms has been challenging for students from all ages, but particularly mature students. This study sought to explore mature students’ experiences of university during the COVID-19 pandemic through the lens of the Community of Inquiry (CoI) model. Utilising a qualitative approach, twenty semi-structured interviews were conducted with students from across the University of Warwick. The interviews were conducted by a staff member and a mature student during the pandemic and four themes were identified: Adapting to online learning, relationships, external factors and response of the university. This research study has identified that some participants responded well to the emergency situation, others had caring responsibilities which impacted on their studies but that peer relationships and collaborative learning is key to their success.

## Introduction

The journey through education differs for many, but mature students are often those who have missed out on formal education in the early parts of the lives, seeking additional qualifications to increase employment chances, or to start a new livelihood (Burke, 2007; James & Boeren, 2019). The number of mature students entering higher education over the last 15 years has significantly decreased (Callender & Thomson, 2018), and the decline accelerated with the introduction of higher tuition fees in 2012, and then again in 2015 (Fraser & Harman, 2019). In the United Kingdom (UK) the definition of a mature student is that the individual must be over 21 years of age when they commence undergraduate study or over 25 years old when they commence postgraduate study (Higher Education Statistics Agency, 2020).

Committing to starting a programme in a Higher Education Institution (HEI) setting is a considerable risk for mature students, where there are often financial, family, caring and work commitments to contend with (Busher & James, 2017). This can also be coupled with fears over failure due to poor prior experiences of education (Brine & Waller, 2004).

Higher education can be transformational for mature students as they seek to navigate their way to a ‘better life’ (Broadhead, 2018), but it is widely acknowledged that mature students are often those who can be described as being from underrepresented or disadvantaged groups (Boeren et al., 2017; OFFA, 2017). The impact of the challenges facing mature students can be seen by the fact that they are more likely to drop out of their courses and have poorer degree outcomes than their younger peers (OfS, 2020), although some authors have argued this is ‘normally due to personal or financial factors rather than academic failure’ (Lucas & Ward, 1985 cited in Richardson & King, 1998).

## COVID-19 Impact

Since early 2020, the COVID-19 pandemic has forced many HEIs to pivot their pedagogical practice to meet the needs of students (Tadesse & Muluye, 2020). While there is evidence that developing countries have been affected more severely (UNESCO, 2020), university students in the UK have had to quickly adapt to online teaching and learning (Burki, 2020). Distance, blended and online learning moved away from being a relatively small, niche approach to university study, to being the norm, as universities sought to cope with the changing demands of the COVID-19 pandemic (El Refae et al., 2021). As campuses closed across the UK, the absence of face-to-face teaching (or extremely limited periods of interaction) meant that virtual learning platforms (VLEs), online communication platforms and other web-based learning technologies (Bower & Torrington, 2020) became increasingly vital as the emergency situation unfolded (Narayan et al., 2020). The University of Warwick responded as many other HEI’s in the UK, in the early part of 2020, the vast majority of learning moved online. Later in the pandemic, the University moved to a period of blended learning, and towards the end of 2022 academic year, the vast majority of learning moved back to face-to-face learning, this study is situated in the middle of the pandemic (summer 2021). It should also be acknowledged that in other parts of the world there were differing restrictions in academic institutions during the pandemic (United Nations, 2020).

There are long-established routes for mature students to access HEI qualifications at a range of institutions. This includes Further Education Colleges (FECs), traditional universities, but also other distance learning universities, which are almost exclusively online (most commonly in the UK, the Open University). The use of digital learning, and platforms, is not a new concept for some mature students (Staddon, 2020) and blended approaches, where students interact with each other online (Canning, 2010) have been found to be beneficial in increasing students’ confidence. The growing use of technology enhanced learning (TEL) over the last 20 years demonstrates that mature students have becoming increasingly used to accessing some of their learning online (Henderson et al., 2015). Whilst there is a perception that mature students are more concerned about using technology in their learning (Staddon, 2020), others note that we should be cautious in making overly simplistic arguments about older students engaging in the use of digital tools (Laming et al., 2019; Pearce, 2017).

This paper seeks to understand the impact of the COVID-19 pandemic on a group of mature students in one university in the UK. The research seeks to understand how students reacted to the pandemic pedagogy (Schwartzman, 2020) and what they perceived as the challenges, and opportunities, that the forced enclosure of the university presented to them.

## Theoretical Framework

This study applied notions of Community of Inquiry (CoI) as a framework for analysis of the mature student experience of online learning during the pandemic. The model was seen by the researchers as an appropriate lens to investigate the mature student experience as it enabled an exploration of a community of learners through specific time frame, and an unprecedented turn to new ways of studying (Dumitru, 2012).

**Figure 1.**
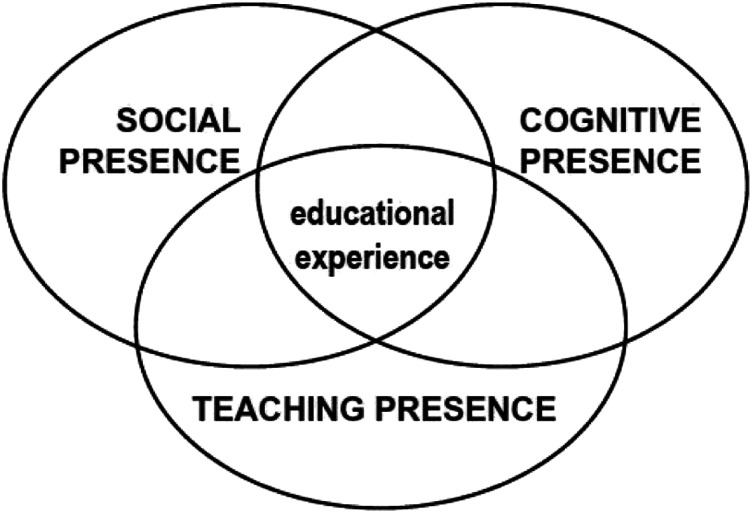
Community of Inquiry Model.

Community of Inquiry is rooted in Dewey’s (1938) concepts of social constructivism (Castellanos-Reyes, 2020), in which learning is considered as a social activity whereby we construct knowledge in conjunction with, and alongside, teachers and peers (Swan, 2019). This model of studying the complexities of online learning is borne out of research by Garrison et al. (1999), which ‘outlines critical dimensions that influence student-learning experiences in an online environment’ (Kim & Gurvitch, 2020). The CoI model sees the online learning experience as having three overlapping components (see Figure 1) which are social presence, cognitive presence and teaching presence (Richardson et al., 2012).

Garrison (2007: 63) states that social presence is ‘the ability to project one’s self and establish personal and purposeful relationships’. Social presence has also been linked to an ability to feel connected (Rettie, 2003; Tu & McIsaac, 2002) and the capacity to identify with a group to enable personal relationships (Garrison, 2016). Cognitive presence is defined by Garrison, et al. (1999: 89) as the extent to which participants are ‘able to construct meaning through sustained communication’, which should be collaborative and involve the ability to express one’s thoughts, but also time to think and listen to others (Garrison, 2017). The last component of the model is teaching presence, which includes not only how online learning is organised and put together, but also ‘the facilitation of learning, and direct instruction’ (Swan, 2019: 61).

Community of Inquiry has been adapted over the last 20 years and is used in many different contexts and studies of online learning, but it is also not without its critics. Previous researchers have argued that there should be additional ‘presences’ within the model, but as yet there is no agreement to what these should be (Kozan & Caskurlu, 2018). Castellanos-Reyes (2020) notes that most studies have been conducted in American and Canadian settings, while Annan (2011: 42) argues that CoI ‘does not adequately inform the development of online education theory and practice’. Furthermore, CoI’s application to students’ online learning during the COVID-19 pandemic is still relatively under-researched (Banerjee et al., 2020; Patwardhana et al., 2020), as well as an understanding of the mature student experience during this unusual academic year (Anderson, 2021).

However, as Kim and Gurvitch (2020, p. 397) state ‘CoI originated as a framework for assessing the quality of online learning experiences’ and this has been the most unfamiliar HEI experience in years for both staff and students, as they adapt to an emergency situation (Patwardhan et al., 2020). Community of Inquiry will provide a framework to explore the mature student experience during the pandemic, by applying the three CoI presences in relation to the qualitative interview data (Kucuk, & Richardson, 2019).

## Participants

A purposive sampling strategy was employed for this research study (Creswell & Creswell, 2017). The sample was sought from students who identified as being mature and who were studying at the University of Warwick. The researchers sought participants from across the University, and they were recruited from a variety of means, including email, digital newsletters and internal departmental communications. Mature students volunteered to take part in the study and were given a book voucher for their time. All students who responded to the call for participants were asked to take part in the study. The participants were from a range of backgrounds, ages and experiences, and they were all enrolled on either undergraduate or postgraduate programmes, either in a full or part-time capacity. The criteria for selection meant that the students had to be over 21 years old (for undergraduate study) or over 25 years old (for postgraduate study) to take part in the interview process. The students studied a range of subjects, some were part of departments such as the Centre for Lifelong Learning (CLL) and others were in disparate subjects such as Psychology or Linguistics (see Table 1).

**Table 1.** Study Participants.

Name*	Level of study	Subject	Age bracket
Kathy	Undergraduate	BA Social Studies	46 to 55
Carolyn	Undergraduate	BA Social Studies and Counselling	46 to 55
Wendy	Undergraduate	BA Social Studies	36 to 45
Timothy	Undergraduate	BA Social Studies	56 to 65
Kaylee	Undergraduate	History	26 to 35
Stacey	Undergraduate	History and English	56 to 65
Lena	Undergraduate	BA Early Childhood Studies	46 to 55
Diane	Undergraduate	BA Social Studies (Philosophy major)	36 to 45
Nathan	Undergraduate	Biomed, Life sciences	18 to 25
Cameron	Postgraduate	IER PhD	56 to 65
Kelly	Undergraduate	Person Centred therapeutic relationship degree	46 to 55
Anna	Undergraduate	Social Studies CLL	56 to 65
Ivy	Undergraduate	CLL – BA in Social Studies (majoring in Sociology)	26 to 35
Monica	Undergraduate	CLL Health and Social Policy	36 to 45
Rebecca	Postgraduate	BA Education	46 to 55
Paul	Postgraduate	MSc in Intercultural Communications for Business and the Professions	70+
Carmen	Postgraduate	MSc Intercultural communications for business & professions - Applied Linguistics	36 to 45
Marc	Undergraduate	BA (Hon) Social Studies	56 to 65
Lela	Other	PhD, History	36 to 45
Joyce	Undergraduate	PAIS	18 to 25

*Please note these are pseudonyms.

Twenty participants volunteered to be interviewed, 75% of the interviewees were female, and the remainder (25%) were male. The interviewees were between the ages of 23–71, 20% were under 35, 75% were between 36 and 65 and 5% over 70 years of age. The interviews were conducted over a 3-month period in 2021 and were conducted on the online platform, Microsoft Teams.

## Methodology

The research study used semi-structured interviews, with a predetermined set of questions, but with a degree of flexibility to ensure that issues and matters that were important to the participants could be explored fully (Given, 2008). The interviews were conducted by two researchers, one of whom was a member of staff at the university (and a former mature student) and the other an undergraduate at the university (who was a current mature student). We concur with Mann and Stewart (2001) who argue that mutual trust is essential for developing good rapport with interviewees. To this end, we explained our positions as a former, and current mature student, to participants before the interviews took place. This was in part to demonstrate our receptivity, but also to recognise the often sensitive nature of participants reflecting on their own personal lives and educational journeys. The interviews were also conducted with constructivist principles, acknowledging that interviewees and interviewers ‘share the goal of understanding the complex world of the lived experience from the point of view of those who live it’ (Schwandt, 1994, p. 118).

The study was undertaken after receiving ethical approval from the University of Warwick Ethics Committee, which is bound by a Research Ethics Code of Practice, and complies with the UK Data Protection Act 1998 and the Data Protection Principles.

Before the interviews were agreed upon, each participant was given a participant information leaflet, which outlined the process and also a consent form. These measures ensured that the research was conducted within ethical guidelines (BERA, Ethical Guidelines for Educational Research, 2018).

## Data Analysis

The qualitative data from the interviews was first transcribed and then processed using the data analysis software Nvivo, the data was coded using a thematic approach (Charmaz, 2014). The analysis of the data was formed from the words the participants spoke during the interviews, by using a systemic approach of revisiting the data and creating codes (Vaismoradi et al., 2016). Through the data analysis process, 23 codes were identified, which then led to four emergent themes. There was no preconceived hypothesis, but instead, the coding and subsequent development of themes was borne out of emerging patterns in the data set (Smith & Davies, 2010).

The themes were then applied to the overarching theoretical framework, CoI. The components that make up the CoI model: social presence, cognitive presence and teaching presence (Garth-James & Hollis, 2014) are examined in relation to the views that the students expressed about their learning online during the pandemic.

The semi-structured interviews were conducted with current University of Warwick students, but their identity is protected in these findings, with anonymised verbatim quotations and the use of pseudonyms (Given, 2008). The aspiration of using these measures was to provide reassurance for the participants, but also to create a safe space where they felt able to talk freely without any concerns about negative effects on their future university lives (Broadhead et al., 2020).

## Findings

Some of the participants who took part in this research study had started their studies during the pandemic (thus had only known their courses to be blended or online), others had experiences of face-to-face delivery at the start of their courses but then had recently had to pivot to working the majority of the time online. However, all participants had at least 12 months’ experience of the online, blended learning approach. The University of Warwick, as with many other universities in the UK, continued to offer a small element of face-to-face delivery (usually seminars) where lockdown rules and governmental rules allowed (Nerantzi, 2020). It is acknowledged that this was, in many respects, a new experience for both university staff and the students, but also that it was, and still is, considered an emergency temporary situation (Nordmann et al., 2020).

It should also be noted that this study concentrated on the mature student experience, and that the challenges facing older students can substantially differ from their younger peers (Laming et al., 2019). It is arguable that these issues were exemplified during the pandemic with parents facing additional challenges as UK schools closed down and 90% of children were home schooled (Office for National Statistics, 2021).

### Adapting to Online Learning

For many of the participants, the move to online learning was difficult, and there was a perception that the younger students made the process of interaction during synchronous sessions restrictive:

Most of the problem we had on our lectures and seminars was (that) people didn’t put their cameras on and didn't interact. And actually as a mature student that just, we just didn't do that. I was always like, camera on, interact, put my hands up, I'm like well if you’re not gonna talk I’m going to ask the questions ‘cause I wanna know the answers. (Rebecca)

Most of the other student participants shared the frustration of trying to study online, and felt that it was not the same positive comparable experience to face-to-face delivery:

I’ve not learned as much. Uh, you know, trying to learn on Teams is really hard, especially when most of the 19 and 20 year old students. They don't want their cameras on, so you can't……yeah, it's a poor experience. It's a very poor imitation. (Timothy)

The issue of being unable to see the other students face-to-face within the online environment was a key concern for the mature students. It was felt by a large number of the participants in this study that when students had their cameras on it led to a more conducive and open discussion forum, rather than seeing a blank screen:

One (module) was so painful because by this time we're obviously all on, Zoom or Teams, the other students wouldn't have their mics on or the cameras on and so going forward into year two, I’m not doing another module with younger students because I just did not enjoy the experience. (Kelly)

The reasons for students having their cameras (and microphones) off during online synchronous classes is a complex matter, with some arguing that it should not be a requirement due to issues of privacy and comfort in what is already a stressful and anxious pandemic situation (Castelli & Sarvary, 2021). However, most participants in this study felt that it impeded their ability to have a social presence, and did not support the feelings of social and emotional connections with their classmates (Richardson et al., 2012). Other studies (Whiteside et al., 2017) have demonstrated how the virtual meetings where people can see each other on screen increases the immediacy and intimacy that individuals feel.

Examples of positive teaching presence was also discussed by some of participants, where the staff had clearly put a lot of time and effort into adapting their teaching materials to ensure they would work in the online space, as Ivy describes:

I had another module where the tutor, oh my goodness, I don't how many hours she must spend working on this module, it is mind-blowing. Like to get it all online. It was so interactive. She put so much thought and effort in. Yeah, that it was absolutely incredible.

The remodelling of entire degree courses to work online has been a challenge for many staff who teach in universities, with a realisation that designing synchronous and asynchronous learning is time consuming (Nordmann et al., 2020). There was a recognition from some participants that moving online has actually had benefits, particularly for mature students:

I would never have been able to do this Master’s part-time, had it not been a pandemic, because my classes were like Monday 10a.m. till 11a.m., Tuesday… like they were spread across the week and I would literally never, I would have had to take a year off work to do it full time, which I couldn't have done.

The time to study is often a key concern for mature students, who often have a wider range of responsibilities, such as parenting and caring commitments, than their younger peers (Norton et al., 1998). The pivot to online, blended learning meant that students could access their subjects when it suited them:

You know it’s been great to have online lectures and watch them in bits in my own time, and also I’ve really enjoyed working with some of the younger students on the programme. (Paul)

Some students advocated their studies staying online, with Ivy advocating for a continued ‘hybrid approach’:

I think it is really important just to be able to access the papers online. Makes such a difference because there’s five floors in that library. And I’ve got 20 minutes until I’ve got go and pick the kids up from school, like… I can’t, I don't have the skills, knowledge, or time to scour the whole flippin’ place.

While it is perhaps a generalisation to say that mature students need more technical support than others (O’Driscoll et al., 2010), a student gave an example of where her peers had provided a social presence to ensure that she could continue with her online lesson:

I had to say, can you just help me I’m a bit of a technophobe. I don't know how to do this, and they were really, really kind with me. This is my other students, even though they weren't chatty or communicative, they were very, you know. Just let me be a bit more there. A bit more patient with me trying to sort out the technology.

Participants gave further examples of peer support where they used digital technology to communicate with each other, outside of the formal networks provided by the University.

Well, I socialize within my group….we have WhatsApp and we have various things on Facebook where we keep in contact. You know outside of the classroom. (Marc)

Having that support, having some, you know, in a little WhatsApp group if you're worried about something, you could ask, what do you think about this? Do you know where this is? That was absolutely brilliant. We all brought something to that group and helped each other, and that was really important for me. Going through again, I think I would have struggled quite a lot if it hadn’t been for that. (Rebecca)

These examples of cognitive presence, where students are able to exchange knowledge, connect with each other and new ideas, were often done on an informal ad hoc basis, outside of the University structures. This informal peer-to-peer feedback helps contribute to cognitive presence and also supports an increase in social presence (Sun et al., 2017).

### Relationships

The start to University had been unprecedented for some of the mature students who took part in this study. The ability to form relationships, and friendships with other students on their courses had been difficult for the majority of participants. For example, Carmen stated that:

I think it(s) group work really where we missed out on kind of making friends because if I'd been able to come to some of the lectures, even if it only been like once a week, maybe then I would have at least seen everybody, and we’d have been able to hang out after class a little bit and things like that.

Other students who had studied at the University before the pandemic found that they had limited opportunities to interact during this online experience:

I don't think I know anyone this year on my course. No, I mean, I know I don’t know anyone that's on my calls and it’s not, this is just not the same. And yeah, I think it would have been nice to get - and maybe it will come back, it’s probably just a remote working thing, rather than a course thing, hopefully. (Anna)

The development of relationships, and friendships, with peers whilst studying at University can be integral to mature students’ sense of identity (O'Boyle, 2014). When mature students do not form these bonds it can lead to feelings of insecurity and isolation (Stuart, 2006), while social isolation can seriously affect the attainment of students at University (Doman & Roux, 2010). Many of the students who took part in this study had felt they were committing to a face-to-face degree programme, but the pandemic had severely impacted on their ability to converse with their peers, even when they did manage to go into the University:

I did feel though that it made life a bit difficult in terms of bonding with other students and having seminars, because actually saying that, whilst I did go in for some seminars at the beginning of the first term that was actually worse because we were all spread out in a lecture theatre, facing the front with masks on and there was just no backward and forward, and nobody wanted to speak. (Rebecca)

Rapidly designing and delivering degree programmes during an unprecedented academic year, with changing government advice, and an emphasis on blended learning has undoubtedly been challenging for academic staff (Hodges et al., 2020). However, to support social presence greater emphasis could be placed on activities online and face-to-face interactions, which encourage students to share their personal experiences, which may be supported by more structured learning activities (Richardson et al., 2017).

### External Factors

It is well understood that mature students have layers of additional responsibilities that other younger students may not (Sutton, 2019), and that these issues have been exemplified during the pandemic as home schooling and home working became the norm (Cheng et al., 2021). Within this study, there were some parents whose experience of University had been impacted by responsibilities outside of the classroom:

Oh God, we struggled… it was just day by day really. You know some days the kids just pretty (much) wouldn't do it. They just wouldn't have it. Some days I got really upset. And some days I just shut the door and my husband would try and entertain them while I got on with some.... It was just, it was massive and a real struggle. (Kelly)

I’m working at honours level now but not very competently truth be told, this has been a bit of a nightmare of a year. Its fine, like I've passed, but it, yeah, not what I wanted really, and I’ve gotta resit for the research project because that was just a bridge too far with home schooling. (Ivy)

Childcare commitments were an issue for a number of mature students, and some expressed concerns about the pressure associated with having to study and care for others:

That element of pressure for mature students to look after children to continue to work and to maintain the same standard at university that they were maintaining before online and not in person, it was a huge ask really. (Kelly)

Some other participants had wider caring responsibilities, which had caused issues:

That the pandemic came at a point where us as a family were already sort of struggling and drowning with various different things with my husband’s health and so it has really impacted. Because it's impacted, you know, and I’m being stretched even thinner because he’s out of action. I'm caring for him, then the kids are at home, and home schooling and running a house and not being able to leave the house and all those different things. (Monica)

So like you know, my parents don’t like require care, like they're still pretty independent. But like in the last couple of years, like I’ve had to deal with, my mom breaking her hip. You know, not healing properly, her being in like incredible pain, and to have a hip replacement during Covid which was a nightmare. (Nathan)

I've got very elderly parents, so I can't afford to, well, I couldn't afford to get Covid myself at my age or pass it on to my parents, and so I'd already spoken to them about my concerns of coming in. (Kelly).

These responsibilities are often taken on by mature students quietly and with a reluctance to share concerns with staff at the University (Marandet & Wainwright, 2013). The combination of face-to-face and online learning approaches was difficult for some mature students to adapt to, coupled with wider obligations. The development of cognitive presence requires space for learners to reflect, explore ideas and have a discourse with fellow students (Gibson et al., 2012), with many of the competing demands placed upon mature students. It could be inferred that the opportunities for collaborative learning were reduced by the restrictions around the external responsibilities faced by mature students.

### Response of the University

Many mature students had not intended to enrol on a course which offered so much online content, and this was an unprecedented shift in an extraordinary situation (Means & Neisler, 2021). As one of the participations (Nathan) stated, ‘starting (University) in a pandemic is not anyone’s optimal situation’. The university fared well in the annual 2021 National Student Survey (NSS) of final year students, where over 3000 students placed Warwick third in the Russell Group and 13th in the UK for overall satisfaction ((University of Warwick, 2021)).

Some students discussed how they accessed support from the university for extra time, or extensions on their deadlines, which were generally well received:

I just think they have tried to move heaven and earth in the last couple of years to accommodate and to make sure that everybody……they're trying to level out the playing field. (Kelly)

I have had a deadline moved because of all the children home during lock down and there was no questions asked about that, it was just granted to me. (Rebecca)

This degree of flexibility was arguably essential when students faced mounting pressures to deal with multiple competing demands on their time. Mature students often assume more responsibility for their studies (Briedenhann, 2007), often using skills they have learnt during other periods of their lives. Similarly, in CoI classrooms (Stover & Ziswiler, 2017) where relationships with staff at the University can lead to a sense of caring within their course communities (Stodel et al., 2006).

Other students had different experiences, and felt more could have been done:

I think a lot of academics this year they seem to...there’s a lot of like, oh, it’s really difficult for us this year. It's like, it's really difficult for us too, and it feels like there’s a lot of, like, we're being asked to cut them some slack and we don't get any slack. If anything, we are expected to work harder. (Nathan)

But I feel like they really needed to step up even more with Covid and they haven’t really and then they should be putting more (support) ‘cause this is going to have a knock on effect for years to come. (Monica)

Other students discussed further support that could have been given, perhaps more of a ‘safety net’ (Cameron), with a recognition that mature students ‘need different levels of support and different levels of help, and we shouldn’t pretend that, you know, everybody is the same’ (Anna). Teaching presence during blended learning approaches is vital if students are to feel supported and confident in seeking help (Martzoukou & Kemp, 2016). There have been considerable efforts by teaching staff in organising and delivering blended learning, which even outside of a worldwide health crisis can take considerable effort (Anderson, 2021). Teaching presence is significant in supporting students to achieve, even outside of the online learning environment (de la Varre et al., 2011) therefore the University emphasis on the whole student experience is important, even in an emergency pivot to blended learning.

The desire to ‘retain some traditional ways of learning’ (Lam, 2015) may have impacted on some mature students’ ability to feel part of the learning community, where they felt like they belonged to an institution or part of their course peer group (Tu & McIsaac, 2002). The sudden move to blended learning meant that some students were not necessarily taking part in the education experience they were originally intending to do, with the inherent biases that some feel towards online or distance education as being a poor substitute for face-to-face learning (Oyarzun et al., 2021). Multiple participants reiterated the desire for moving back to learning at the university, for example:

I think we need to get back on campus or back to face-to-face (learning) because if we wanted to do remote learning we would all signed up for the Open University. (Timothy)

Whilst there was a clear desire from most students to get back to face-to-face learning, a few students were also keen to ensure that a ‘hybrid approach’ (Ivy) continued as there were clear benefits for those with complex, competing commitments, as Kaylee stated: ‘when they’re mature, they’ve got more responsibilities’. The mixture of online and face-to-face delivery was seen by many participants as a way of fitting in University study around a busy life ‘it’s been great to have online lectures and watch them in bits in my own time’ (Paul).

## Discussion

This research concentrated on the mature students’ experience of studying during the pandemic. The findings of this research demonstrated that although the students had differing experiences, in many respects they had all succeeded as they were continuing in their respective studies, and whilst some had used the University’s deferral systems (e.g. mitigating or extenuating circumstances), none had left the University. By using the CoI as a framework, it has been possible to examine where the three categories of presence were within their blended study experiences (Swan et al., 2009). From the data analysis, four themes were identified such as *Adapting to online learning*, *Relationships, External factors and Response of the university*. Perhaps, unsurprisingly, we found that the unexpected pivot to online and blended learning has been challenging for some students, whilst for others it has had advantages they may not have envisaged. For the majority of participants who took part in this study, the move online was understood as essential due to the pandemic, but perhaps not welcomed.

From a technological perspective, the move to blended learning was not found to be difficult for most of the mature participants, and the use of online platforms and working practices were relatively straight forward (Burgess, 2008). Unlike in other studies, there were no major issues identified with accessing the internet, or IT equipment (Coyner & McCann, 2004), rather there was identification of the loneliness and isolation which can be found from studying online (Luyt, 2013). With more time available for course design and preparation, further consideration could be given to ways in which social presence can be built more effectively for mature students (Kear et al., 2014). It could also be hypothesised that due to mature students expecting, and being more familiar, with traditional face-to-face learning, the move to synchronous class sessions with videoconferencing platforms was frustrating for some of the participants when they could not physically see, or hear their peers (Reich et al., 2020). This hindered their ability to feel like they were part of the group, which in turn resulted in frustration and feelings of the impersonal, which did not endear the platforms to the older students and demonstrated a lack of social presence (Kear, 2010). Most of the students used informal social media platforms to create a cognitive presence with each other during the pandemic. This use of technology outside of the University’s sphere helped them to feel supported, and was important in their ability to stay connected with their peer group.

This research also discovered that students understood where staff had worked to provide teaching presence, and the effort they had put into pivoting the learning to a blended approach (Shea et al., 2006). Conversely, there was also a feeling amongst some participants that more empathy should have been given to the students during the pandemic, and that at times the University could have done more to support them during these uncertain times (Aini et al., 2020). This type of support could have been extended to further instruction on how to take part in blended learning, as well as more opportunities for online social activities between students, which may have helped alleviate some of the feelings of loneliness.

The multiple responsibilities that the mature students faced during the pandemic, from caring for others to home schooling, arguably acted as barriers to engagement at times and decreased the ability of the students to feel ‘group cohesion (social presence), and greater engagement with course materials and assignments (cognitive presence)’ (Majeski et al., 2018). As the findings of this research have demonstrated, the mature student experience is often multifaceted and multi-layered, with levels of responsibility that is often unseen and underestimated.

## Conclusion

This study has found that mature students at the University of Warwick found aspects of the emergency pivot to online learning challenging, but they, similar to many other learners across the country, adapted and saw some positive outcomes in what was doubtlessly one of the most difficult times to study at University. The complexities of mature student experience meant that they had more caring commitments to contend with, in comparison to their younger peers. Although some struggled with isolation and fears of loneliness, mature students valued the human, personal contact with their peers and staff, and were looking forward to a more ‘conventional’ University experience. However, the blended learning approach had clear benefits for those with competing demands on their time, and as we move back to more traditional modes of learning, lessons should be learnt about the more valuable aspects of online learning for mature students.

For 20 years, CoI has been used as a model for analysis of different modes of the online learning experience (Castellanos-Reyes, 2020). Whilst this study has helped to illuminate the mature student experience through the lens of CoI, there are limitations to the research. The participants all came from one institution, and their experiences cannot speak for others who may study elsewhere. Similarly, the mature students who took part in the research all had access to the internet and IT equipment – this may limit our understanding of those who were not afforded such privileges.

The findings of this study warrant further exploration of the longer-term impacts on this underrepresented group (Laming et al., 2019) because the effects of the last two academic years may well cause further issues, but also benefits to mature students. The use of CoI has been a useful framework to analyse the student experience of blended learning during a pandemic (Shastri & Clark, 2021). Staff at all differing levels of education have worked hard to rapidly adapt their courses to work within blended or online approaches (Nordman et al., 2020). Only over the next academic years will we see whether the lessons and practice learnt will continue to be applied in the future.

Mature students place a significance on collaborative learning and their personal development where they can share their understandings with each other (Zhang, 2020), it is hoped that as we move to a post-pandemic world, they can find these opportunities in both the online and traditional learning spaces.
